# Mechanistic Study on the Antidepressant-Like Effect of Danggui-Shaoyao-San, a Chinese Herbal Formula

**DOI:** 10.1155/2012/173565

**Published:** 2012-08-09

**Authors:** Zhen Huang, Qing-Qiu Mao, Xiao-Ming Zhong, Zhao-Yi Li, Feng-Mei Qiu, Siu-Po Ip

**Affiliations:** ^1^College of Pharmaceutical Sciences, Zhejiang Chinese Medicine University, Hangzhou 310053, China; ^2^School of Chinese Medicine, The Chinese University of Hong Kong, Hong Kong

## Abstract

Danggui-Shaoyao-San (DSS), a famous Chinese herbal formula, has been widely used in the treatment of various diseases. Previous studies have shown that DSS produces antidepressant-like effect in rodents. This study aims to investigate the mechanism(s) underlying the antidepressant-like action of DDS. The results showed that DSS treatment significantly antagonized reserpine-induced ptosis in mice. In addition, DSS treatment significantly increased sucrose consumption in chronic unpredictable stress- (CUS-) treated mice. DSS treatment also markedly attenuated CUS-induced decreases in noradrenaline and dopamine concentrations in mouse brain. Furthermore, DSS treatment significantly reversed CUS-induced increase in serum malondialdehyde (MDA) content and decrease in serum superoxide dismutase (SOD) activity in mice. The results suggest that the antidepressant-like activity of DSS is probably mediated by the modulation of central monoamine neurotransmitter systems and the reduction of oxidative stress.

## 1. Introduction

Depression is a prevalent psychiatric disease affecting the quality of life of many people. Based on latest available data from the World Health Organization, depression is expected to become the second leading cause of disease-related disability by the year 2020. It is generally believed that monoamine neurotransmitters including serotonin (5-HT), noradrenaline (NA), and dopamine (DA) are involved in the pathogenesis of depression, and most antidepressant drugs exert their action by elevating monoamine neurotransmitters concentrations [1, 2]. Oxidative stress, which is defined as a disturbance in the balance between the production of reactive oxygen species (ROS) and antioxidant defense systems, has also been shown to play a role in the pathogenesis of neuropsychiatric disorders [3, 4]. Furthermore, preclinical studies have demonstrated that the inhibition of oxidative stress may contribute to the therapeutic effects of some antidepressant drugs [5–8].

Danggui-Shaoyao-San (DSS) is a traditional herbal medicine which is widely used in China, Japan, and Korea. DSS, the herbal formula, is first recorded in “JinKuiYaoLue” and it consists of six medicinal herbs (Table 1). The herbal drug was traditionally used to relieve menorrhalgia and other abdominal pains of women [9]. Recently, a clinical study has showed that DSS is effective in treating insomnia [10], which is a symptom and predictor of depression [11]. Preclinical studies have showed that DSS possesses antioxidative, antithrombotic, cognitive enhancing, and neuroprotective effects [9, 12, 13]. Moreover, a previous report suggested that DSS treatment could significantly decrease immobility time in the forced swim test in mice [14]. The herbal preparation was also effective in improving chronic stress-induced behavioral alterations in rats, which were related to the central arginine vasopressin system [14]. However, the action mechanism for the antidepressant-like effect of DSS is still not fully elucidated. In this study, we aim to explore different antidepression mechanisms of DSS by investigating its effect on the monoamine neurotransmitter systems. In addition, the effects of DSS on brain monoaminergic neurotransmitter and serum antioxidant status were examined in an animal model of depression [15, 16].

## 2. Materials and Methods

### 2.1. DSS Preparation

**Figure 1:**
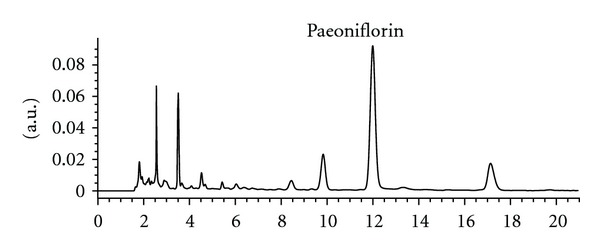
High-performance liquid chromatography analysis of DSS.

All the crude drugs of DSS were purchased from Zhejiang Provincial Hospital of Traditional Chinese Medicine (Zhejiang Province, China). They were identified and authenticated by Associate Professor KR. Chen, College of Pharmacy, Zhejiang Chinese Medicine University, where voucher specimens (number 100925) had been kept. Aqueous extract of DSS was prepared as following procedure of Kou et al. [12]. In brief, six medicinal materials were mixed in proportion and were macerated for 1 h with eight volumes of distilled water and then decocted for 1.5 h. Next, the cooled extract was filtered. The extraction procedure was repeated twice. The extracted fractions were pooled and concentrated using a rotary evaporator. The yield of the extract was 29.32% on dry weight basis. DSS extract contained 1.43% of paeoniflorin as analyzed by high-performance liquid chromatography (Figure 1). HPLC analytical conditions were as follows: a Waters Nova-Pak C18 HPLC column (4.6 × 250 mm) was used for the separation. Separation was achieved by an isocratic elution with a mobile phase consisted of acetonitrile and 0.04% phosphoric acid (16 : 84, v/v) at a flow rate of 1.0 mL/min. The eluate was monitored by a diode array detector at wavelength of 230 nm.

### 2.2. Chemical Reagents

5-Hydroxytryptamine (5-HT), noradrenaline (NA), and dopamine (DA) were purchased from Sigma-Aldrich (St. Louis, MO). Paeoniflorin was purchased from National Institute for the Control of Pharmaceutical and Biological Products (Beijing, China). Other reagents were analytical grades made in PR China.

### 2.3. Animals

Male ICR mice weighing 20–25 g were obtained from the Laboratory Animal Services Center, Zhejiang Chinese Medicine University, Hangzhou, Zhejiang. The animals were individually maintained on a 12 h light/dark cycle (lights on at 6:00 a.m., lights off at 6:00 p.m.) under controlled temperature conditions (22 ± 2°C) and given standard food and water *ad libitum*. They were allowed to acclimatize for seven days before use. All experiments conformed to the guidelines of the “Principles of Laboratory Animal Care” (NIH publication number 80-23, revised 1996) and the legislation of the People's Republic of China for the use and care of laboratory animals. The experimental protocols were approved by the Animal Experimentation Ethics Committee of Zhejiang Chinese Medicine University. Effort was made to minimize the number and suffering of the animals.

### 2.4. Reversal of Reserpine-Induced Ptosis in Mice

The animals were randomly assigned into five groups of eight individuals: control group (physiological saline), control plus DSS-H group (3 g/kg), reserpine plus vehicle group (physiological saline), reserpine plus DSS-L group (1.5 g/kg), and reserpine plus DSS-H group (3 g/kg). DSS and physiological saline were given intragastrically daily between 9:30 to 10:30 a.m. for seven days. The doses used in the present study were selected on the basis of Xu et al. [14] and our preliminary tests. Sixty minutes after the last dose, mice in reserpine groups were injected intraperitoneally with 2.5 mg/kg of reserpine. The degree of ptosis of each animal was recorded at 60 min after the injection of reserpine. For the evaluation of ptosis, mice were placed on a shelf (20 cm above the bench top). The degree of ptosis was rated according to the following rating scale: 0, eyes open; 1, one-quarter closed; 2, half closed; 3, three-quarters closed; 4, completely closed [17].

### 2.5. Chronic Unpredictable Stress (CUS)

Mice were randomly assigned into four groups of eight individuals: control group, CUS plus vehicle group (physiological saline), CUS plus DSS-L group (1.5 g/kg), and CUS plus DSS-H group (3 g/kg). The CUS procedure was performed as described by Mao et al. [6], with a slight modification. Briefly, mice in stressed groups were individually housed and exposed to the following stressors once daily for 21 days: 24 h food deprivation, 24-h water deprivation, 7 h cage tilt (45°), 24 h exposure to a foreign object (e.g., a piece of plastic), 1 min tail pinch (1 cm from the end of the tail), 21 h soiled cage (200 mL water in 100 g sawdust bedding), and overnight illumination. DSS and physiological saline were given intragastrically 30 min before each stressor once every day for 21 days. Control (unstressed) animals were undisturbed except for necessary procedures such as routine cage cleaning.

### 2.6. Sucrose Preference Test

Sucrose preference test was carried out 1 day after CUS starting at 09:30 am. The test was performed as described previously [18] with minor modifications. Briefly, 72 h before the test, mice were trained to adapt 1% sucrose solution (w/v): two bottles of 1% sucrose solution were placed in each cage, and 24 h later 1% sucrose in one bottle was replaced with tap water for 24 h. After adaptation, mice were deprived of water and food for 12 h, followed by the sucrose preference test, in which mice were free to access to two bottles containing 100 mL of 1% sucrose and 100 mL of tap water, respectively. After 1 h, the volumes of consumed sucrose solution and water were recorded, and sucrose preference was calculated as calculated using the following formula:

(1) Sucrose  preference =Sucrose  consumptionWater  consumption+sucrose  consumption×100%.

### 2.7. Blood and Tissue Collection

Twenty-four hours after the behavioral test (2 days after CUS), between 09:30 am to 11:30 am, mice were sacrificed by decapitation to obtain venous blood samples on ice. Different groups of mice were used for each time point. Serum was separated by centrifugation at 4000 g for 10 min at 4°C and stored at −80°C until assay. Following blood collection, their whole brains were quickly removed, frozen in liquid nitrogen and stored at −80°C until assayed.

### 2.8. Measurement of Monoamine Neurotransmitter Levels

Brain 5-HT, NA, and DA levels were measured by HPLC coupled with electrochemical detection method as described previously [19]. Briefly, each frozen tissue sample was homogenized by ultrasonication in 200 Al of 0.4 M perchloric acid (solution A). The homogenate was kept on ice for 1 h and then centrifuged at 12,000 g (4°C) for 20 min. The pellet was discarded. An aliquot of 160 *μ*L of supernatant was added to 80 *μ*L of solution B (containing 0.2 M potassium citrate, 0.3 M dipotassium hydrogen phosphate, and 0.2 M EDTA). The mixture was kept on ice for 1 h and then centrifuged at 12,000 g (4°C) for 20 min again. Twenty *μ*L of the resultant supernatant was directly injected into an ESA liquid chromatography system equipped with a reversed-phase C18 column (150 4.6 mm I.D., 5 *μ*m) and an electrochemical detector (ESA CoulArray, Chelmstord, MA, USA.). The detector potential was set at 50, 100, 200, 300, 400, and 500 mV, respectively. The mobile phase consisted of 125 mM citric acid-sodium citrate (pH 4.3), 0.1 mM EDTA, 1.2 mM sodium octanesulfonate, and 16% methanol. The flow rate was 1.0 mL/min. 5-HT, NA, and DA were identified and quantified by comparing their retention times and peak areas to those of standard solutions. The contents of 5-HT, NA, and DA were expressed as ng/g wet weight tissue.

### 2.9. Measurement of MDA Level and SOD Activity

Serum MDA level was determined by measuring thiobarbituric-acid reacting substances [20]. Serum SOD was determined based on its ability to inhibit the oxidation of oxymine by O^2−^ produced from the xanthine/xanthine oxidase system [21]. Protein concentration was determined by the Coomassie blue protein binding [22] using bovine serum albumin (BSA) as a standard. The detailed procedures of measurements followed the manufacture instruction in different reagent kits (Nanjing Jiancheng Institute of Biological Engineering, China).

### 2.10. Statistical Analysis

Data are expressed as means ± SEM. Significant differences between means were performed using one-way analysis of variance (ANOVA) followed by Dunnett's test. A difference was considered statistically significant when *P* < 0.05.

## 3. Results

The effect of DSS on reserpine-induced ptosis in mice was given in Table 2. One-way ANOVA showed a significant difference on the mean score of ptosis among groups (*F*(4,35) = 97.9, *P* < 0.01). Treating the animals with DSS-H did not provide any significant change on the mean score of ptosis when compared with the controls. The reserpine injections resulted in a significant increase in the mean score of ptosis in the animals (*P* < 0.01) compared with the controls. Treating the animals with DSS-L or DSS-H significantly decreased the mean score of ptosis in the reserpine-treated mice (*P* < 0.01 and *P* < 0.01, resp.) compared with the reserpine-treated control.

**Figure 2:**
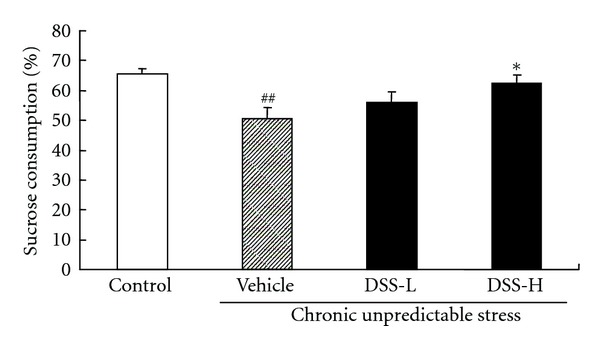
Effect of DSS treatment on the percentage of sucrose consumption in CUS-treated mice. Values given are the mean ± SEMs (*n* = 8). ^##^ *P* < 0.01 as compared with the nonstressed control; *P < 0.05 as compared with the CUS-treated control.

The effect of DSS on the percentage of sucrose consumption in CUS-treated mice was given in Figure 2. One-way ANOVA showed a significant difference on the percentage of sucrose consumption among groups (*F*(3,28) = 6.3, *P* < 0.01). A 21-day CUS exposure significantly reduced the percentage of sucrose consumption in the animals (*P* < 0.01) compared with the control (i.e., non-CUS-treated mice). Long-term treatment with DSS-H significantly increased the percentage of sucrose consumption in CUS-treated mice (*P* < 0.05) compared with the CUS-treated control.

The effect of DSS on brain monoamine neurotransmitter levels in CUS-treated mice was given in Table 3. One-way ANOVA showed a significant difference on the concentrations of 5-HT, NA, and DA in mouse brain among groups (*F*(3,28) = 12.9, *P* < 0.01, *F*(3,28) = 21.6, *P* < 0.01, and *F*(3,28) = 3.7, *P* < 0.05, resp.). A 21-day CUS exposure significantly decreased the concentrations of 5-HT, NA, and DA in mouse brain (*P* < 0.01, *P* < 0.01, and *P* < 0.05, resp.) compared with those of the controls. DSS-H treatment significantly increased the concentrations of NA and DA in brain of CUS-treated mice (*P* < 0.01 and *P* < 0.01, resp.) compared with the CUS-treated control, while DSS treatment did not produce a significant influence on brain 5-HT concentrations.

**Figure 3:**
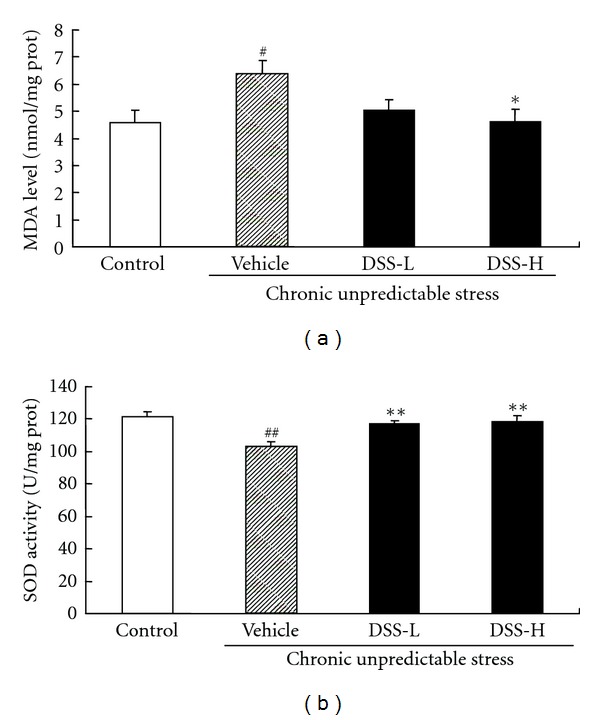
Effect of DSS treatment on serum antioxidant status in CUS-treated mice. The antioxidant status was assessed by measuring MDA level (a) and SOD activity (b). Values given are the mean ± SEMs (*n* = 8). ^#^ *P* < 0.05; ^##^ *P* < 0.01 as compared with the nonstressed control; *P < 0.05; **P < 0.01 as compared with the CUS-treated control.

The effect of DSS on serum antioxidant status in CUS-treated mice was given in Figure 3. The antioxidant status was assessed by measuring MDA level (Figure 3(a)) and SOD activity (Figure 3(b)). One-way ANOVA showed a significant difference on MDA level and SOD activity among groups (*F*(3,28) = 4.1, *P* < 0.05, and *F*(3,28) = 7.6, *P* < 0.05, resp.). A 21-day CUS exposure significantly increased MDA level (*P* < 0.05) and reduced SOD activity (*P* < 0.01) when compared with those of the controls. DSS-L or DSS-H treatment significantly increased SOD activity in CUS-treated mice (*P* < 0.05 and *P* < 0.01, resp.) compared with the CUS-treated control. DSS-H treatment also significantly decreased MDA level in CUS-treated mice (*P* < 0.05) compared with the CUS-treated control.

## 4. Discussion

Several hypotheses have been suggested for the pathological mechanism of depression. The early hypothesis of depression, namely the monoamine hypothesis, supposed that the main symptoms of depression were due to the functional deficiency of brain monoamine neurotransmitters such as 5-HT, NA, and/or DA [23]. Consistent with this view, drugs that are acted by increasing the bioavailability of brain monoamine neurotransmitters, such as tricyclic antidepressants, selective serotonin reuptake inhibitors, and monoamine oxidase inhibitors, are widely used in clinical depression treatment [24]. The reserpine-induced depression is animal model based on the monoamine hypothesis of depression. Reserpine can irreversibly inhibit the vesicular uptake of monoamines, including noradrenaline, dopamine, and 5-hydroxytrytamine. As a consequence, ptosis and hypothermia are observed as the depletion of monoamines stores [25, 26]. These syndromes can be antagonized by major classes of antidepressant drugs. In this study, pretreating mice with DSS for 7 days significantly antagonized reserpine-induced ptosis, suggesting that the antidepressant effect of DSS may be mediated via central monoaminergic neurotransmitter system.

Several studies suggest that CUS can induce behavioral and physiological changes resembling symptoms of clinical depression [8, 15, 16, 27–29] and that CUS-induced depression model can be used for evaluating the efficacy of antidepressants through the sucrose preference test [8, 16, 19, 27–29]. The sucrose preference test is an indicator of anhedonia-like behavioral change [16]. Anhedonia, a core symptom of major depression, is modeled by inducing a decrease in responsiveness to rewards reflected by reduced consumption of and/or preference for sweetened solutions [16]. The results of the present study showed that mice subjected to a 21-day period of CUS consumed less sucrose solution when compared to nonstressed mice, while long-term treatment with DSS significantly suppressed this behavioral change. Although we did not use a conventional antidepressant as positive control in this study, the CUS-induced depression model is generally thought to be the most valuable and steady depressive model in animals and has been successfully established in our previous studies [6, 18].

To further investigate the antidepression mechanisms of DSS, the effects of DSS on the monoamine neurotransmitter systems in CUS-treated mice were studied. Our results showed that CUS caused significant decreases of 5-HT, NA, and DA levels in mouse brain which were consistent with other studies [30–34], while DSS treatment significantly increased the concentrations of NA and DA, but not 5-HT, in brain of CUS-treated mice. Moreover, previous reports have shown that DSS increased the levels of brain monoamine neurotransmitters in normal mice as well as in aged mice [12, 35]. These results suggested that the antidepressant-like effect of DSS may be related to the monoamine neurotransmitter systems, particularly NA and DA systems.

Recent studies have shown that reactive oxygen species (ROS) also play a role in the pathogenesis of depression [3, 4]. Previous studies have demonstrated that chronic stress caused a significant increase in the production of ROS [36]. Excessive ROS can cause damages to the major macromolecules in cells, including lipids, proteins, and nucleic acids, culminating in neuronal dysfunction and depression [8, 37]. MDA, a by product of lipid peroxidation, is produced under oxidative stress. It indicates the oxidative damages of the plasma membrane and resultant thiobarbituric acid reactive substances, which are proportional to lipid peroxidation and oxidant stress [38]. It has been reported that brain MDA level was significantly increased in rodents exposed to chronic stress, which could be reversed by antidepressants [6, 7, 36, 39]. There is an intrinsic antioxidant defense system in cells for scavenging ROS to prevent cellular damage. SOD, one of the most important antioxidant enzymes, has been shown to directly catalyze the transformation of peroxides and superoxide to nontoxic species [40]. Previous studies have showed that chronic stress caused a significant decrease in brain SOD activity in rodents, and antidepressant treatment was found to restore the level of SOD [5, 36]. Consistently, the present study showed that 21-day CUS caused a marked increase in oxidative stress as characterized by excessive MDA production and a reduction in SOD activity. However, DSS treatment attenuated these changes in the CUS-treated mice, suggesting that the antidepressant-like effect of DSS may be related to its antioxidant activity.

It has been shown that oxidation of monoamine neurotransmitters by monoamine oxidase might result in increased radical burden [41, 42]. On the other hand, ROS has been shown to modulate the synaptic transmission, resulting in decreasing DA release [39]. The foregoing findings suggest an association between monoamine oxidation and overproduction of ROS in the pathogenesis of depression. It has been suggested that the antidepressant-like effect of antioxidant is mediated via the monoaminergic system [43]. The present study also suggested a correlation between the monoamine system and oxidative stress in CUS-treated mice. This finding was consistent with previous studies [5, 6, 44].

In conclusion, the antidepressant-like effect of DSS may be mediated by the modulation of central monoaminergic neurotransmitter systems and the inhibition of oxidative stress. Further investigation on the interaction among the component herbs of DSS is warranted.

## Figures and Tables

**Table 1:** Composition of Danggui-shaoyao-san (DSS).

Components	Ratio
(1) Dang gui (*Angelica sinensis* (Oliv.) Diels., root)	3
(2) Bai Shao (*Paeonia lactiflora* Pall., root)	16
(3) Fu Ling (*Poria cocos* (Schw.) Wolf., fungus nucleus)	4
(4) Bai Zhu (*Astractylodes macrocephala* Koidz., root and rhizome)	4
(5) Chuan Xiong (*Ligusticum chuanxiong* Hort., rhizome)	8
(6) Ze Xie (*Alisma orientale* (Sam.) Juzep., rhizome)	8

**Table 2:** Effect of DSS on reserpine-induced ptosis in mice.

Treatment	Ptosis mean score
Control	0.0
Control + DSS-H	0.0
Reserpine + vehicle	2.2 ± 0.4^##^
Reserpine + DSS-L	0.4 ± 0.3**
Reserpine + DSS-H	0.5 ± 0.3**

Values given are the mean ± SEMs (*n* = 8).

^##^ *P* < 0.01 as compared with the control; **P < 0.01 as compared with the reserpine-treated control.

**Table 3:** Effect of DSS treatment on brain monoamine neurotransmitter levels in CUS-treated mice.

Treatment	5-HT (ng/g)	NA (ng/g)	DA (ng/g)
Control	1016.2 ± 43.1	1433.5 ± 66.7	1444.4 ± 59.8
CUS + vehicle	713.2 ± 18.4^##^	1012.2 ± 34.6^##^	1254.8 ± 38.6^#^
CUS + DSS-L	779.0 ± 47.9	1002.7 ± 33.4	1369.8 ± 19.1
CUS + DSS-H	831.0 ± 27.4	1181.8 ± 27.2*	1445.1 ± 56.6*

Brain monoamine neurotransmitter levels were obtained by using HPLC coupled with electrochemical detection method. Values given are the mean ± SEMs (*n* = 8).

^#^ *P* < 0.05; ^##^ *P* < 0.01 as compared with the nonstressed control; *P < 0.05 as compared with the CUS-treated control.
